# Anaphylactic Induction of Lupus Nephritis and Posterior Reversible Encephalopathy Syndrome Following Administration of Depo‐Provera

**DOI:** 10.1155/crii/6631845

**Published:** 2026-08-18

**Authors:** Adaora Ezeani, Sarah Staskiewicz

**Affiliations:** ^1^ Marana Health, Tucson, Arizona, USA; ^2^ Department of Radiology, Cooper University Hospital, Camden, New Jersey, USA, cooperhealth.org

**Keywords:** case report, depot medroxyprogesterone acetate, hypersensitivity reaction, posterior reversible encephalopathy syndrome, systemic lupus erythematosus

## Abstract

Systemic lupus erythematosus (SLE) is a multifactorial autoimmune disease in which environmental and medication‐related exposures may precipitate disease onset or flares in susceptible individuals, which can result in lupus nephritis (LN) and, in rare cases, posterior reversible encephalopathy syndrome (PRES). Immediate hypersensitivity reactions preceding the initial presentation of SLE have rarely been described. We report that a 21‐year‐old Hispanic woman developed airway‐compromising angioedema, encephalopathy, and generalized tonic‐clonic seizures, requiring intubation after her first depot medroxyprogesterone acetate (DMPA) injection. Brain magnetic resonance imaging (MRI) demonstrated PRES with extensive posterior vasogenic edema consistent with PRES with hemorrhagic transformation and bilateral posterior cerebral artery vasoconstriction. Laboratory evaluation revealed acute kidney injury with proteinuria, hypocomplementemia, positive antinuclear and antidouble‐stranded DNA antibodies, and positive SSA antibodies. Kidney biopsy confirmed class IV diffuse LN. Treatment included pulse corticosteroids, seizure prophylaxis, immunosuppressants, and antihypertensive therapy, resulting in neurological and renal recovery. Although causality cannot be inferred, the temporal sequence raises the hypothesis that immune activation associated with DMPA exposure, or hypersensitivity to one of its excipients, may have contributed to the clinical unmasking of autoimmune disease.

## 1. Introduction

One of the basic mechanisms theorized to cause tissue damage in patients with systemic lupus erythematosus (SLE) is the deposition of circulating immune complexes (ICs) in target organs, which activate the complement system, further degrading the tissue [1]. For example, when ICs are deposited into the renal system, a common complication called lupus nephritis (LN) develops. In rare cases, SLE has been associated with posterior reversible encephalopathy syndrome (PRES), a neurological condition consisting of white matter edema that presents with various symptoms such as altered mental status, seizure, headache, and visual changes [1]. The etiology of PRES is not fully understood but may be related to endothelial dysfunction or cerebral hyperperfusion secondary to factors such as renal failure, cytotoxic medications, or autoimmune disorders such as SLE [1, 2]. Here, we present a case of a 21‐year‐old female with PRES and LN as the first manifestation of SLE after administration of Depo‐Provera.

## 2. Case Presentation

A 21‐year‐old Hispanic female with no significant medical history presented to the emergency department (ED) with complaints of facial swelling and worsening generalized edema, headache, chest pain, shortness of breath, dizziness, and blurry vision for 3 weeks. The patient stated that prodromal symptoms, such as headache and dizziness, began after receiving her first Depo‐SubQ Provera or depot medroxyprogesterone acetate (DMPA) followed by facial swelling three to 4 days later. Over the course of three ED visits and one visit to her primary care provider, she was prescribed corticosteroids, cetirizine, lisinopril, and furosemide for hypertension and edema. The past medical history was significant for spontaneous vaginal delivery 1 year prior.

On admission, the patient was hypertensive and tachypneic. She was alert and oriented, with no neurological deficits. She had lateral nystagmus exacerbated by head rotation with a normal fundoscopic exam. Bilateral lower‐extremity edema was also present. Initial laboratory results (Table 1) were significant for elevated blood urea nitrogen (42 mg/dL) and serum creatinine (1.7 mg/dL) and decreased glomerular filtration rate (44 mL/min/1.73 m^2^), total protein (5.3 g/dL), and albumin (2.3 g/dL). While waiting for imaging, she subsequently developed acute neurological deterioration characterized by progressive encephalopathy with agitation and confusion and new‐onset generalized tonic‐clonic seizures. Concurrent worsening tongue, facial, and neck edema resulted in progressive upper airway compromise requiring endotracheal intubation for airway protection and transfer to the intensive care unit (ICU).

**Table 1:** Initial complete blood count, comprehensive metabolic panel, and coagulation panel.

Labs	Value	Reference range
Complete blood count		
WBC	8.2	4.0–11.0 10^3^/uL
RBC	4.58	4.20–5.40 m/uL
Hb	13.8	12.0–16.0 g/dL
Hct	40.4	35.0%–50.0%
Platelet count	168	130–450 k/uL
Comprehensive metabolic panel		
Sodium	140	136–145 mmol/L
Potassium	5	3.4–5.1 mmol/L
Chloride	111	98–107 mmol/L
Carbon dioxide	20	21–31 mmol/L
Creatinine	1.7^a^	0.6–1.3 mg/dL
Blood urea nitrogen	42^a^	7–25 mg/dL
Glucose	110^a^	74–109 mg/dL
Calcium	7.5^a^	8.6–10.3 mg/dL
Glomerular filtration rate	44^a^	≥60 mL/min/1.73 m^2^
Anion gap	9	3–11 mEq/L
Alkaline phosphatase	64	35–105 u/L
Aspartate aminotransferase	17	13–39 u/L
Alanine aminotransferase	8	7–52 u/L
Protein, total	5.3^a^	6.0–8.3 g/dL
Bilirubin, total	0.5	0.2–1.2 mg/dL
Lipase	26	11–82 u/L
Albumin	2.3	3.5–5.7 g/dL
Procalcitonin	0.20	0.00–0.50 ng/mL
Coagulation panel		
Prothrombin time	10.6	9.4–12.6 s
Activated partial thromboplastin time	24.1^a^	25.1–36.5 s
International normalized ratio	0.91	0.90–1.20

^a^Indicates results that are outside the normal reference range.

### 2.1. Hospital Course

In the ICU, the patient was sedated on propofol, started on steroids, seizure prophylaxis and control medication, and continuous EEG monitoring. Empiric antibiotic therapy was started for possible meningitis and aspiration pneumonia. Serial laboratory tests showed anemia with thrombocytopenia, hyperkalemia with metabolic acidosis, and azotemia (Table 1). Urinalysis showed significant hematuria (3+), pyuria (2+), and proteinuria (3+) with granular and hyaline casts, revealing progressive anuric acute renal failure. Lumbar puncture showed mild pleocytosis with elevated red blood cells, segmented neutrophils, protein, and glucose. Infectious workup was negative.

**Figure 1:**
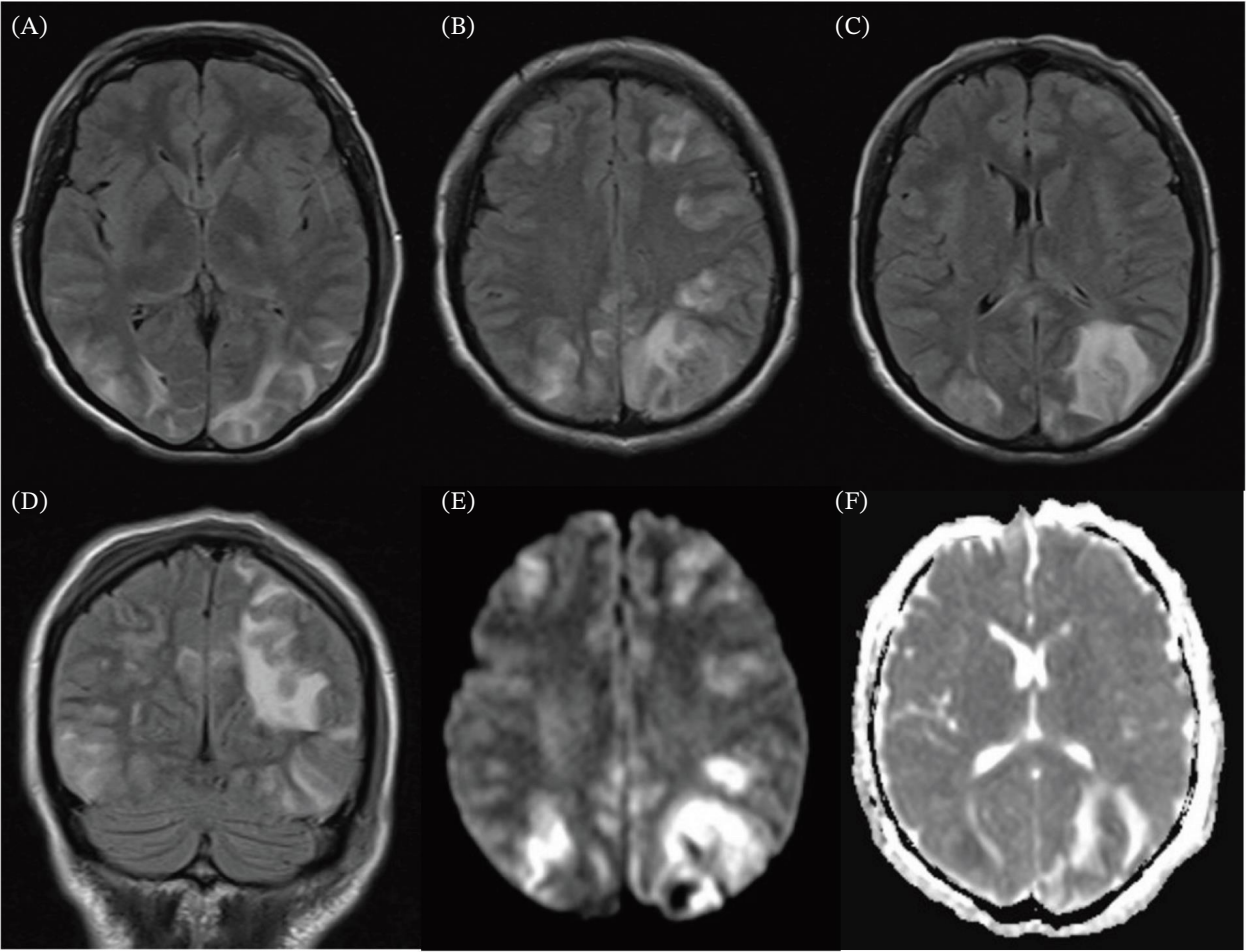
MRI brain with contrast. (A, B) Axial T2 FLAIR shows multiple areas of cortical and subcortical vasogenic edema most prominent in the parietal and occipital lobes and, to a lesser extent, the frontal lobe. (C, D) Axial and coronal T2 FLAIR identified a hyperintense area of acute hemorrhage in the left parietal lobe with surrounding vasogenic edema and mass effect. (E, F) Axial DWI also demonstrates vasogenic edema as a hyperintense signal.

Initial neuroimaging was limited by significant motion artifact related to recurrent seizure activity. Clinically, the patient demonstrated rapid neurological decline with persistent encephalopathy, prompting repeated neuroimaging. Repeat head CT without contrast, as well as brain magnetic resonance imaging (MRI) with angiography (MRA), and brain magnetic resonance venography (MRV), were ordered. MRI demonstrated predominantly posterior subcortical vasogenic edema involving both cerebral hemispheres with superimposed areas of restricted diffusion, indicating cytotoxic edema, a finding associated with more severe disease and a risk of irreversible parenchymal injury (Figure 1). A parenchymal hematoma in the left parieto‐occipital region was also identified, representing hemorrhagic transformation of PRES, a complication reported in 10%–15% of cases [3]. MRA showed focal occlusions of bilateral posterior cerebral arteries (PCAs) proximally with distal reconstitution, a pattern consistent with severe segmental vasoconstriction rather than thromboembolic occlusion. This finding suggests overlap with reversible cerebral vasoconstriction syndrome (RCVS), which has been reported to coexist with PRES in 15%–30% of cases and may represent a shared spectrum of cerebral vascular dysregulation [4]. MRV showed no evidence of cerebral vein thrombosis. The saline contrast echocardiogram was negative for thrombus, indicating that bilateral PCA occlusions were likely due to vasoconstriction. Given the concurrent vasogenic edema and bilateral PCA vasoconstriction, the patient was started on nimodipine, and serial transcranial Dopplers were ordered to track the degree of vasoconstriction. Repeat head CT showed no progression of hemorrhage or edema. She did not experience any seizures after ICU admission. On day 4, she was extubated and placed on low‐flow oxygen.

Autoimmune serological workup (Table 2) was ordered. The initial antinuclear antibody test was negative, but the repeat was positive (1:640 on human epithelial type 2 cells, homogeneous pattern). Other significant results included low complement levels (C3: 52 mg/dL and C4: 6 mg/dL), strong positive anti‐dsDNA IgG antibody by indirect immunofluorescence assay (IFA) (1:2560), and positive antibodies to extractable nuclear antigen SSA‐60/Ro60 (127 AU/mL). The results supported the diagnosis of SLE with PRES. Methylprednisolone was increased to 1000 mg every 8 h for 3 days, and then she was transitioned to oral prednisone 60 mg daily. Renal biopsy demonstrated diffuse LN, class IV, and she was started on mycophenolate mofetil (MMF) 1000 mg po twice a day. Considering the patient’s clinical improvement, she was transferred to the inpatient floor and subsequently discharged on prednisone, MMF, and famotidine for GI prophylaxis, levetiracetam for seizure prophylaxis, and antihypertensive medications with rheumatology, neurology, and nephrology follow‐up.

**Table 2:** Immunoserology panel.

Labs	Value	Reference range
ANCA IFA titer	<1:20	<1:20
ANCA IFA pattern	—	—
ANA direct	Positive^b^	Negative
ANA pattern	homogeneous + SSA/Ro^b^	—
ANA titer	1:640^b^	<1:160
dsDNA^a^ Ab, IgG	>300^b^	0–24 IU
dsDNA^a^ Ab, IgG IFA titer	1:2560^b^	<1:10
β2‐glycoprotein 1 Ab, IgM	<10	≤20 SMU
β2‐glycoprotein 1 Ab, IgG	<10	≤20 SGU
C1 esterase inhibitor, functional	83	≥68%: normal 41%–67%: equivocal ≤40%: abnormal
Cardiolipin, IgA	<10	≤11 APL: negative 12–19 APL: indeterminate 20–80 APL: low to moderately positive ≥81 APL: high positive
Anticardiolipin, IgG	<10	≤14 GPL: negative 15–19 GPL: indeterminate 20–80 GPL: low to moderately positive ≥81 GPL: high positive
Cardiolipin, IgM	<10	≤12 MPL: negative 13–19 MPL: indeterminate 20–80 MPL: low to moderately positive ≥81 MPL: high positive
Complement C3	52^b^	90–180 mg/dL
Complement C4	6^b^	10–40 mg/dL
Myeloperoxidase Ab	20	≤19 AU/mL: negative 20–25 AU/mL: equivocal ≥26 AU/mL: positive
Antiphosphatidylserine, IgG	0	0–15 GPS
Antiphosphatidylserine, IgM	0	0–21 MPS
Antiphosphatidylserine, IgA	0	0–19 APS
Ribonucleic protein (Sm/RNP) Ab, IgG	13	≤19 units: negative 20–39 units: weak positive 40–80 units: moderate positive ≥81 units: strong positive
Serine proteinase 3 (PR3), IgG	0	≤19 AU/mL: negative 20–25 AU/mL: equivocal ≥26 AU/mL: positive
SSB/La Ab, IgG	31	≤29 AU/mL: negative 30–40 AU/mL: equivocal ≥41 AU/mL: positive
SSA‐52/Ro52 Ab, IgG	12	≤29 AU/mL: negative 30–40 AU/mL: equivocal ≥41 AU/mL: positive
SSA‐60/Ro60 Ab, IgG	127^b^	≤29 AU/mL: negative 30–40 AU/mL: equivocal ≥41 AU/mL: positive

Abbreviations: Ab, antibody; ANA, antinuclear antibody; ANCA, antineutrophil cytoplasmic antibody dsDNA, double‐stranded DNA; IFA, immunofluorescence assay; IgA, immunoglobulin A; IgG, immunoglobulin G, IgM, immunoglobulin M.

^a^Positivity for anti‐dsDNA IgG Ab is a diagnostic criterion of SLE. Specimens are initially screened by enzyme‐linked immunosorbent assay (ELISA). If ordered as reflex, positive ELISA results (>24 IU) will be reflexed to a highly specific IFA titer (*Crithidia luciliae* indirect fluorescent test [CLIFT]) for confirmation.

^b^Indicates results that are positive or outside the normal reference range.

Four days after discharge, the patient was readmitted for facial swelling and generalized edema and treated with spironolactone and IV furosemide. She was also found to have severe anemia (hemoglobin 6.6) and received one unit of packed red blood cells and intravenous iron. MMF was increased to 1250 mg twice daily, and she was discharged on spironolactone 25 mg and furosemide 40 mg with specialty follow‐up. Outpatient labs showed normal complement and anti‐dsDNA levels. Edema improved, leading to the discontinuation of spironolactone, reduction of furosemide, and addition of potassium supplementation for hypokalemia. The patient later returned to the ED with recurrent hand and leg swelling, received IV methylprednisolone, had MMF increased to 1500 mg twice daily, and had prednisone reduced to 40 mg daily. Due to GI side effects, MMF dosing was adjusted to 1500 mg in the morning and 1000 mg at night. She developed a perifollicular petechial rash attributed to vitamin C deficiency and was advised supplementation. Prednisone was tapered to 10 mg daily, and furosemide and potassium were discontinued.

## 3. Discussion

PRES is a rare complication of SLE, occurring in only 0.7%–1.4% of cases [5, 6]. Renal involvement is present in 25%–50% of patients with SLE [7] and is reported to be a strong indicator for developing PRES. LN with PRES as the first manifestation of SLE is not common and has not been widely reported in the literature. In addition, there have only been a few reports detailing anaphylaxis to DMPA [8–10].

SLE is a multisystem autoimmune disease that occurs most frequently in young women. Disease pathogenesis involves dysregulation of the innate and adaptive immune systems as well as end‐organ injury from autoantibodies. Aberrant activation of the innate immune cells such as macrophages, dendritic cells, and neutrophils leads to activation of autoreactive T and B cells, creating autoantibodies to nucleic acid‐bound antigens. Autoantibodies form ICs, signaling immune cells to produce proinflammatory cytokines. Furthermore, ICs can deposit in tissues and trigger complement fixation, leading to inflammation‐mediated injury [11, 12]. This can result in a measurable increase in disease activity or flare, if they reach a specific threshold [11]. This patient seemingly remained quiescent for years, including an uncomplicated pregnancy 1 year prior.

SLE flares can affect multiple organ systems; however, the kidneys are typically the primary target, resulting in LN, a type of autoimmune glomerulonephritis [12]. PRES is another complication of SLE, though the pathophysiology is not well understood. Hypotheses include cerebral vasoconstriction causing brain infarcts, cerebral autoregulation failure leading to vasogenic edema, and endothelial damage and blood–brain barrier disruption resulting in fluid and protein transudation in the brain [13]. Therefore, features of SLE activity such as hypertensive urgency or emergency, antiphospholipid antibodies, or renal failure due to LN may be causative factors [13].

Lupus flares can begin in response to a number of stimuli, such as stress, infections, and certain medications, resulting in immunologic activity that can exacerbate symptoms [11]. In this case, the trigger may have been the active ingredient, medroxyprogesterone acetate, or the excipients, such as macrogol/polyethylene glycol (PEG) in DMPA, which have been reported to cause hypersensitivity reactions such as tachycardia, hypertension, and angioedema [9, 10, 14, 15]. For example, medroxyprogesterone acetate, as an antigen, promotes T helper 2 cell development, regulating IgE synthesis and allergic reactions, and directly acts on mast cells or basophils, leading to histamine release, leukotriene production, and cytokine secretion, culminating in systemic anaphylaxis [16]. Concerning PEGs, different mechanisms have been proposed, but diagnostic testing (e.g., intradermal tests, oral provocation, basophil studies, and histamine release tests) strongly suggests an IgE‐mediated mechanism [17]. However, PEG‐specific IgE has not yet been directly identified, largely due to technical challenges in assay development [18].

Due to its low abundance and inability to activate complement, IgE has not received attention in the pathogenesis of SLE; however, IgE‐containing ICs have been detected in the serum of SLE patients and autoreactive IgE antibodies directed to most SLE auto‐antigens, particularly dsDNA, in patients with LN [19]. It may also be a reasonable predictor of active disease as levels of anti‐dsDNA IgE have been correlated with the SLE Disease Activity Index (SLEDAI), which assesses disease activity and complement fractions [20]. These findings suggest a pathogenic role of autoreactive IgE in LN; however, the pathway is unclear, and alternative mechanisms may be involved.

Distinguishing a DMPA‐triggered flare of latent SLE from drug‐induced lupus remains difficult. In this case, the presence of serologic and clinical features more characteristic of idiopathic SLE, including low complement levels, elevated anti‐dsDNA antibodies, renal involvement, and neuropsychiatric manifestations, favored a diagnosis of SLE flare compared to classic drug‐induced lupus. In this case study, the temporal relationship between DMPA administration and the patient’s subsequent clinical deterioration suggests a possible role for DMPA in precipitating the SLE flare. Unfortunately, confirmatory tests (e.g., skin prick testing, enzyme‐linked immunosorbent assays, and leukocyte histamine release) to demonstrate immunoresponsiveness were not performed. Therefore, a definitive causal relationship between DMPA and the reported reaction could not be established, and lupus flares may occur spontaneously or in response to other unrecognized triggers. This limitation should be considered when interpreting the findings. Prospective studies are needed to determine whether hypersensitivity reactions to DMPA or its excipients, particularly PEG, can trigger SLE flares. Analysis of lupus registries and pharmacovigilance databases could also help identify flare patterns temporally related to DMPA use. Finally, translational research examining the immunomodulatory effects of DMPA on innate and adaptive immune pathways may identify biologic mechanisms linking hypersensitivity responses to SLE flare.

## 4. Conclusion

This case illustrates an unusual presentation of SLE with PRES and LN as initial manifestations following the administration of DMPA. It suggests a potential hypersensitivity reaction that may have triggered an SLE flare. This underscores the importance of considering medication‐induced exacerbations in patients with autoimmune disorders. In addition, this case highlights the necessity of heightened awareness and timely management to improve patient outcomes in young women presenting with unexplained neurological and renal symptoms.

## Author Contributions

Conceptualization, data curation, formal analysis, project administration, resources, writing – original draft: Adaora Ezeani. Resources, writing – review and editing, supervision: Adaora Ezeani and Sarah Staskiewicz. Software, visualization: Sarah Staskiewicz.

## Funding

No funding was received for this research.

## Consent

Written informed consent was obtained from the patient for the publication of their medical information, including clinical details and images, with all identifiable features removed to protect their privacy. A copy of the written consent is available for review. This study is exempt from an institutional review board approval.

## Conflicts of Interest

The authors declare no conflicts of interest.

## Data Availability

The data that support the findings of this study are available upon request from the corresponding author. The data are not publicly available due to privacy or ethical restrictions.
